# Novel Rank-Based Statistical Methods Reveal MicroRNAs with Differential Expression in Multiple Cancer Types

**DOI:** 10.1371/journal.pone.0008003

**Published:** 2009-11-25

**Authors:** Roy Navon, Hui Wang, Israel Steinfeld, Anya Tsalenko, Amir Ben-Dor, Zohar Yakhini

**Affiliations:** 1 Agilent Laboratories, Tel Aviv, Israel; 2 Agilent Laboratories, Santa Clara, California, United States of America; 3 School of Computer Science, Tel Aviv University, Tel Aviv, Israel; 4 Computer Science Department, Technion, Haifa, Israel; Victor Chang Cardiac Research Institute (VCCRI), Australia

## Abstract

**Background:**

microRNAs (miRNAs) regulate target genes at the post-transcriptional level and play important roles in cancer pathogenesis and development. Variation amongst individuals is a significant confounding factor in miRNA (or other) expression studies. The true character of biologically or clinically meaningful differential expression can be obscured by inter-patient variation. In this study we aim to identify miRNAs with consistent differential expression in multiple tumor types using a novel data analysis approach.

**Methods:**

Using microarrays we profiled the expression of more than 700 miRNAs in 28 matched tumor/normal samples from 8 different tumor types (breast, colon, liver, lung, lymphoma, ovary, prostate and testis). This set is unique in putting emphasis on minimizing tissue type and patient related variability using normal and tumor samples from the same patient. We develop scores for comparing miRNA expression in the above matched sample data based on a rigorous characterization of the distribution of order statistics over a discrete state set, including exact p-values. Specifically, we compute a Rank Consistency Score (RCoS) for every miRNA measured in our data. Our methods are also applicable in various other contexts. We compare our methods, as applied to matched samples, to paired t-test and to the Wilcoxon Signed Rank test.

**Results:**

We identify consistent (across the cancer types measured) differentially expressed miRNAs. 41 miRNAs are under-expressed in cancer compared to normal, at FDR (False Discovery Rate) of 0.05 and 17 are over-expressed at the same FDR level. Differentially expressed miRNAs include known oncomiRs (e.g miR-96) as well as miRNAs that were not previously universally associated with cancer. Specific examples include miR-133b and miR-486-5p, which are consistently down regulated and mir-629* which is consistently up regulated in cancer, in the context of our cohort. Data is available in GEO. Software is available at: http://bioinfo.cs.technion.ac.il/people/zohar/RCoS/

## Introduction

Gene expression profiling is commonly applied to identify differences between classes of cell types, as manifested in differentially expressed genes [1]–[4]. A typical dataset comprises tens of samples in which the expression levels of thousands of genes are measured. In classified expression data the set of samples is partitioned into different subsets or classes based on prior knowledge, such as normal samples vs. tumor samples or samples of different cancer types. Similarly, it may be partitioned into different conditions, different stages, or different therapy related categories. Most of the current data analysis literature focuses on considering the entire dataset in the process of identifying differentially expressed genes. Various types of genomic variation are significant and often ignored confounding factors in differential expression studies. For example, in Shyamsundar et al. [5] the authors survey messenger RNA expression level variation in normal human tissues, showing the potential confounding effects of inter-tissue variation.

It would be valuable to identify statistically significant differences in various samples that can be reliably attributed to the specific biological state, such as cancer or disease, instead of individual biological variations, as stated above. In many situations, there is opportunity for serial collection of tissue or blood from a patient, experimental animal or cell line [6], [7]. However, many current analysis techniques do not exploit the unique relationships within such data. In other cases, class or patient variability can mask differential expression and needs to be addressed. In this study we analyze matched samples to investigate tumor vs. normal differential expression, which is consistent for multiple tumor types, and describe suitable and robust statistical methods that support this investigation.

Currently, hundreds of microRNAs (miRNAs) have been identified in humans. These are short (usually about 22-nt) noncoding regulatory RNA molecules and their sequences are published in the Sanger miRBase [8]. miRNA expression profiling has been recognized to provide valuable biological information with potential to complement or supersede mRNA profiling [9]. miRNAs regulate target genes at the post-transcriptional level and play important roles in development as well as in cancer [9]–[11] and in other human diseases, including heart disease [12]–[14], schizophrenia [15] and psoriasis [16]. miRNAs are highly differentially expressed in different tissue types [10]. Therefore, to identify miRNA differential expression due to specific conditions we need to minimize the confounding effect of the above tissue dependent differential expression.

Our goal in this study is to identify miRNAs that are consistently differentially expressed in multiple cancer types. To avoid tissue type variability and to measure cancer related differential miRNA activity in each type separately; we use a matched sample dataset consisting of 32 microarray measurements representing 28 matched tumor and normal samples. We use microarrays containing probes for 799 miRNAs to profile miRNA expression in these samples.

Our motivation in seeking miRNAs with consistent differential expression in multiple cancer types stems from the existing knowledge that many biological processes are common to different types of cancers. In particular, several genes are known to be universally differentially expressed across multiple cancer types. The most obvious example is p53. p53 was first discovered in 1979 and since then numerous studies indicated its involvement in multiple cancer types. The importance of regulated activity of intact p53 in preventing tumor formation is indicated by the presence of mutations in the p53 pathway in nearly all cancers [17], [18]. Another example of a universal cancer related protein is p16. This gene resides on chromosome 9 and was found to be mutated or deleted in multiple cancer types [19]–[22]. These are only two specific examples, amongst a large variety of cellular processes that are universally associated with cancer.

Previous studies on the role of miRNAs in cancer include Lu et al. [9] who performed a tumor vs. normal cross-tissue analysis using bead-based flow cytometry technology in a non-paired manner. This study showed that miRNAs are sufficient to accurately classify cancer tissues according to their embryonic lineage, giving global characteristics of miRNA expression in cancer. Another study, by Volinia et al.[10], described microarray measurement of 228 miRNAs in 540 samples (363 cancer and 177 normal) from 6 different tissue types. In addition to producing miRNA signatures, the authors reported some miRNAs that are consistently over or under expressed, but there was no detailed statistical benchmarking for the consistency of miRNA differential expression. The authors state that when clustering their data in an unsupervised manner, the samples cluster based on the tissue types, irrespective of the disease status, reflecting the high variation of miRNAs when comparing tissue types. This reinforces our assertion above, that points to miRNA inter-tissue-type basal variation as a confounding factor when seeking to measure miRNA cancer differential expression. Several other studies focus on miRNAs in specific cancer types. For example, mir-15 and mir-16 are frequently deleted and/or downregulated in B-cell chronic lymphocytic leukemia [23], miR-143 and miR-145 show decreased expression in colorectal neoplasia [24], and miR-155 is up-regulated in human B cell lymphomas [25].

To support our research goals we have developed statistical methods that address characterizing distributions of random variables that arise from comparing matched samples. In our case we compute differential expression in every tumor type and then statistically assess its prevalence in our dataset. Our methods are based on discrete order statistics – the k-dimensional vector that is obtained by drawing k independent numbers uniformly in 1…N and then sorting them resulting vector. While the distribution of order statistics over continuous state spaces is well characterized, this is not the case for discrete sample spaces as repeats may then occur with positive probability. Computing distributions related to discrete order statistics was addressed in [26]. For our needs we define random variables over discrete order statistics, fully characterize their distributions and then apply the methods to the biological data to assess statistical significance.

To summarize, the contribution of this paper consists of:

- Rigorous characterization of the distribution of order statistics over a discrete state set as well as of related random variables. This distribution is highly applicable in analyzing matched data in a non parametric setup. We also compare our methods to paired t-test and to the Wilcoxon Signed Rank test.
- A dataset with matched tumor normal samples representing a repertoire of 8 tumor types. This set is unique in its emphasis on minimizing the tissue type and patient related variability through the use of normal and tumor samples from the same patient.
- By applying the novel statistics described above to our matched sample dataset we validate known oncomiRs and describe several novel cancer-universal differentially expressed miRNAs. It should be noted that this stated universality is only substantiated, within the context of this study, for the 8 types represented here.

## Methods

The starting point for analyzing the results of a gene or miRNA expression profiling study is the *expression raw data matrix*. When describing the methods we use the word “gene” but “miRNA” can be used interchangeably. This matrix is typically the output of several pre-processing steps such as normalization and filtering performed on the raw measurement data.

Typically, data analysis of expression profiles starts with the identification and the statistical assessment of genes that are differentially expressed when comparing various classes represented in the cohort. Many current gene scoring methods consider all expression values of a given gene. These are partitioned into two or more populations according to the studied classification. Differences between the resulting subsets of numbers are assessed using various statistical methods. Gene scoring methods fall into two broad categories – parametric methods, and non-parametric (distribution free) methods. Parametric methods assume a certain distribution for the expression values of every gene within each given class (e.g. cancer or normal) and then score genes according to how separate the class specific distributions are. Examples of such methods are the standard *t-test* [27] and the *Gaussian Error* score [28]. Distribution free scores, in contrast, are not based on parametric assumptions. These include the *Kolmogorov-Smirnov* score [29], and the *Wilcoxon Rank-Sum* test [30] as well as the *Information* score [31] and *Threshold-Number-of-Misclassifications* (*TNoM* in short) [31]. The latter nonparametric methods were applied to gene expression and other genomic and genetic data in several studies, as in [2], [32]–[35].

This work is concerned with additional and potentially more relevant information that can be inferred when the expression data is coming from several patients and when all classes were measured for each patient. For example, samples before and after treatment for the same patient. Another example is tumor and normal samples from the same tissue of each patient, a design utilized in this work. The scores we develop take into account the degree to which a gene separates two classes in a large majority of patients. The interpretation is that a gene is relevant to the underlying biology if it is highly differentially expressed for most of the patients. In addition, we attach a significance level (p-value) to each relevance score level. The p-value is the probability to get this level or better, at random, as described below in further detail. Rigorous statistical analysis is instrumental in confidently identifying genes that sharply separate sample classes and thus in pointing at promising research directions. Partial variants of the methods described in this paper were employed in [6] and in [36]. It is particularly important to work with matched statistics when analyzing miRNA expression data, as basal level for these may be highly variable, especially in distinct tissues [10].

In this section we describe the statistical methods in high generality. Specific embodiments, in the context of consistent tumor versus normal miRNA differential expression, are described in the Results Section.

### Rank Consistency Score (RCoS)

The Rank Consistency Score (RCoS) is a differential expression score for 2 classes that takes patient matching into account.

We call the two classes Class A and Class B. We first compute the differential expression between the two classes for every patient (or subject or subset) *k = 1…r* and for every gene *g*. The differential expression can be calculated using different methods and the method chosen depends on the design of the study and on the number of samples for each patient. Differential expression scores include: fold change, Gaussian error score, *t*-test, TNoM and other methods. Often the number of samples for each patient and class is 1, so simple fold change is used.

Next, we rank all the genes per patient according to their differential expression between class A and class B. For every gene *g* we compute its rank for the *k*-th patient: *R_k_(g)* – this is a number between 1 and *N*, where *N* is the total number of genes. The gene *g_top_* for patient k is the one most over-expressed in Class A relative to Class B. It is ranked first and we set 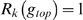. The rank of the gene most under-expressed in the Class A relative to the Class B is *N*.

Our goal is to find genes with consistently high ranks (of differential expression between class A and class B) across all patients. For every gene *g*, we define the rank consistency score *S(g;r)* as the normalized maximal rank of this gene among all patients, i.e.
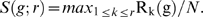



In other words, the rank of gene *g* for all patients is no worse than *S(g;r)·N*.

**Figure 1:**
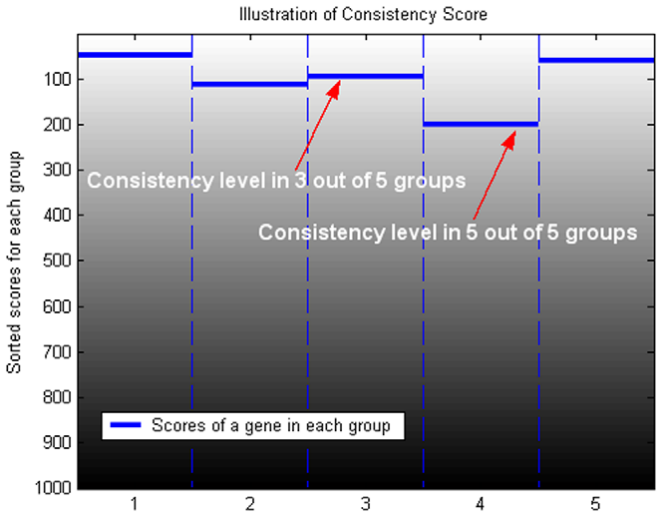
Illustration of Rank Consistency Score. In each of the 5 patients/groups in this example, ranks of the genes change from 1 to 1000. Each column represents a ranked list for one group. The gene chosen for the example has the worst among 5 groups rank of 200. Therefore, its rank consistency is score 200/1000 = 0.2; its rank consistency score in 3 out of 5 patients is 95/1000 = 0.095 as indicated by the arrows.

For greater flexibility in defining consistency we allow outliers, and compute the rank consistency scores *S(g;m)* for *m* out of *r* patients. In this case for each gene we order its ranks and then the score *S(g;m)* corresponds to the normalized *m*-th smallest rank:
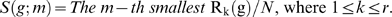



We call the m out of r rank consistency score, *S(g ;m)*, the *m/r* RCoS. We will sometimes refer to the *r/r* RCoS simply as RCoS. Figure 1 illustrates the definition of various *m* out of *r* rank consistency scores. Pseudo-code for calculating the m/r RCoS is available at Text S1.

The above analysis will identify genes that are over-expressed in Class A compared to Class B. To find genes over-expressed in the Class B we can perform the same analysis, reversing the ranked list.

To evaluate the statistical significance of any observed value of RCoS we estimate the probability of obtaining the value s, or better, in random data drawn according to a null model. This probability is the *p-value* corresponding to this level *s*, under the prevailing null model. The *p*-values for RCoS and for its variants considered in this paper are computed under the assumption of independence of patients and of uniform distribution of ranks among genes within each patient. These two assumptions define the underlying null-model.

To compute the m/r RCoS *p*-value at s, we compute the probability of a gene ranking in the top s fraction of the list, in at least m patients. Let *V* be an *r*-dimensional random vector with entries drawn independently and uniformly in *1,…,N*. We are interested in the probability of the *m*-th smallest entry in *V* being smaller than *sN*. It is given by:
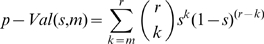



### Minimum Rank Consistency Score (minRCoS)

When working with larger sample sets the question of how many outliers to allow (which m to choose) arises. A possible principled solution is to calculate the m/r RCoS p-value for all possible values of m and choose the value of m with the best p-value. This p-value must of course be corrected for multiple testing. In this section we define the minimal-rank-consistency score, and show how to efficiently characterize its distribution, enabling the calculation of p-values (with no further need for multiple testing correction). We first describe the calculations and then analyze its total time complexity.

For any number *N*>0, we denote the set of ranks {1,..,*N*} by [*N*]; Let [*N*]^r^ represent the set of vectors of length *r*, where each entry is from [*N*]. We use *V* to denote a random vector uniformly distributed over [N]^r^.

Given a vector 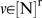 we denote the *m*-th smallest number in *v* by *v*_<m>_. That is, 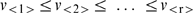. Given an index 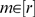, and a rank 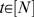, we denote by *β*(*m*,*t*) the probability that *V_<m>_* will equal *t* or less. Note that *β(m,t)* is the p-value, at s = *t/N*, of m-out-of-r rank consistency score defined previously, and can be efficiently computed as shown in the previous section.

We define the *minimal rank consistency score* of a vector *v*, denoted by *mRCoS*(*v*), by 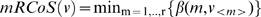. In words, *mRCoS*(*v*) is the best (minimal) rank consistency p-value, where *m* varies from *1* to *r*. *mRCoS*(*V*) is therefore a random variable taking values in [0,1]. We now compute the exact p-value associated with *mRCoS*(*V*) at a given value, p:
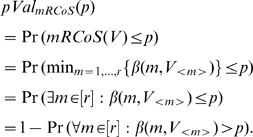



Given 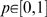, and an index 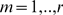, define 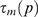 to be the minimal rank *t* such that 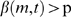. Note that since we can efficiently compute *β(m,t)* for all 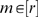 and 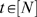, we can efficiently “invert” *β(m,t)* and compute *τ_m_*(*p*). Note that 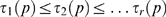. Using the above notation we have:
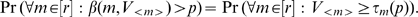



Given a constant ranks vector *C*, we say that a vector *v*∈[*N*]*^r^* is *C*-*bounded* if 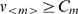 (for all *m* = 1,..,*r*). In words, all sorted entries of *v* are larger (or equal to) the corresponding entries of *C*. For example, the vector *v* = <3,2> is bounded by 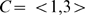, since 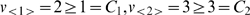.

The total number of vectors in [*N*]^r^ that are *C*-bounded is denoted by *B*(*N*,*r*,*C*).

For example, for 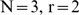,
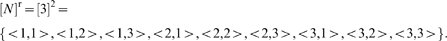



The set of vectors bounded by 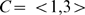 is 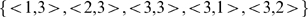, and therefore 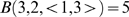.

By the definition of *B*(*N*,*r*,*C*), since *V* is chosen uniformly at random, we get 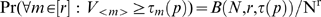, where *τ*(*p*) denotes the vector 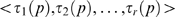. Therefore, we have reduced the problem of computing a p-value for the minimal-rank-consistency score to the combinatorial problem of efficiently computing how many vectors in [*N*]^r^ are bounded by a given vector 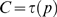.

### Computing *B*(*N*, *r*, *C*)

Given two integers, *N*, *r*, and a vector *C*, we want to compute *B*(*N*,*r*,*C*), the number of *C*-bounded vectors in [*N*]^r^. For each vector *v* we define two properties: *t(v)* and *k(v)*.

- *t(v)* is the maximal entry of *v*. That is, 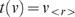. Note that *t(v)* can assume the values 1 through *N*.
- *k(v)* is the number of entries in *v* whose value is strictly smaller than *t(v)*. Note that *k(v)* can assume the values 0 through *r*−1.

These two properties can be used to partition [*N*]^r^.

We denote the set of all *C*-bounded vectors for which 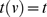 and 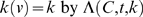. Note that these sets are indeed disjoint, and that their union covers all *C*-bounded vectors. By using 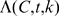 we can compute *B*(*N*,*r*,*C*), summing over all possible values of *t* and *k*:
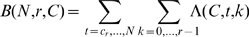



As there are only N*r such sets this would yield an efficient procedure to compute *B*(*N*,*r*,*C*). We use a dynamic programming approach to compute all N*r values.

Let *C*(1..*k*) be the first *k* elements of *C*, that is 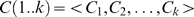. We note that in a vector 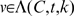 the (r-k) largest ranks equal *t*. Therefore, to compute 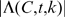 we need only determine the positions within *v* of the *k* smallest values, and their actual values, such that they are all strictly smaller than *t*, and are C(1..k) bounded:
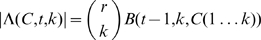



We now use the following dynamic programming procedure to compute the number of *C*-bounded vectors:
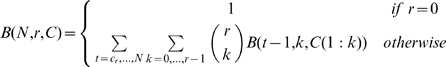



This enables us to efficiently compute the minRCoS p-value:
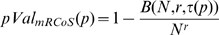



There are a total of N*r dynamic programming steps needed to calculate B(N,r,C). In each step, calculating B(t,k,C) requires summing over t*k values of B. In total the complexity of the dynamic programming procedure to compute B(N,r,C) is therefore O(N^2^*r^2^). To compute 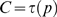 we need to perform a maximum of r*N RCoS p-value calculations, each one taking O(r). Therefore, the complexity of the minRCoS p-value calculation for a given p is O(N^2^*r^2^).

### Samples, Experimental Protocol and Data Pre-Processing

The data were collected from adjacent tumor-normal total RNA samples purchased from Ambion/ABI (FirstChoice® Human Tumor/Normal Adjacent Tissue RNA). The matched pairs of tumor and normal RNAs were from 14 different patients and 8 different cancer types. Tissue samples were of various embryonic lineages: One pair from breast, lymphoma, and prostate; two pairs from liver, ovary, testes and lung; and 3 pairs from colon. Technical replicates were performed for the ovary and testes samples, thus a total of 32 microarray data were used for this study.

For each microarray measurement, 100ng total RNAs were labeled with Cy3 using T4 RNA ligase per Agilent miRNA Micorarray Systems Protocol v1.5. The labeled RNA samples were hybridized onto Agilent miRNA microarray (Agilent Human miRNA Microarray kit V2 - G4470B) for 21 hours at 55C. The arrays contain probes for 723 human and 76 human viral miRNAs from the Sanger database v.10.1. The arrays were then washed at room temperature and scanned to produce the hybridization signals (Agilent miRNA Micorarray Systems Protocol v1.5). The arrays were scanned with extended dynamic range at 5 and 100% PMT using the Agilent scanner (model G2565AA).

Agilent's Feature Extraction software version 9.5.3.1 was used to generate GeneView files [37]. These files contain the processed signals for each of the 799 miRNAs on the array. For each miRNA, expression values (gTotalGeneSignal) below the noise level (gTotalGeneError) were replaced by the value of the corresponding total gene error. All samples were then normalized to have the same 75^th^ percentile value. The raw and normalized data have been deposited in NCBI's Gene Expression Omnibus [38] and are accessible through GEO Series accession number GSE14985 (http://www.ncbi.nlm.nih.gov/geo/query/acc.cgi?acc= GSE14985). All data is MIAME compliant. The normalized data are also available in Table S1.

## Results

We applied rank consistency scoring methods to data collected in a study of miRNA expression profiles in cancer related samples. Data collected in this study consisted of paired samples of tumor and normal origins. Each pair of samples was taken from different parts of the same tissue in 14 different patients and 8 different cancer types: breast, colon, liver, lung, lymphoma, ovary, prostate and testis. The matched pairs of samples enable us to focus on changes in miRNA expression levels that result from the cancer process and to minimize the confounding effect of inter-individual and inter-tissue variability.

The goal of the study was to identify miRNAs universally differentially expressed in cancer using the statistical methods and measurements described above.

We computed the tumor vs. normal differential expression of each miRNA in the data in four different ways: TNoM [31], non paired t-test, paired t-test and minRCoS. For the first three methods, signals were log transformed and in cases where more than one patient exists per cancer type the median was used. The TNoM and unpaired t-test were computed for non-paired comparison of all tumor samples to all normal samples. For the paired t-test the cancer type matching was used.

For the different variants of RCoS (m/r RCoS and minRCoS), fold change was calculated for each miRNA and patient by dividing the tumor signal by the normal signal. In cancer types where more than one patient exists (2 or 3 patients) the median of the fold changes was used. This was done to preserve the patient matching (within the same cancer type) in our data. For each cancer type the miRNAs were then ranked according to these values to generate the ranked lists needed as the input to all the RCoS variants. The application of the general framework described in the Methods section to our dataset therefore leads to the following semantics:

- Class A and class B are tumor and normal.
- r = 8.
- If for a miRNA, denoted g, we have, for example, 6/8 RCoS(g) = 0.2 for over-expression in tumor vs. normal, then this miRNA is ranked amongst the top 20% of miRNAs over-expressed in tumor vs. normal, for at least 6 out of the 8 different tumor types. Obviously, similar interpretations hold true for other values of m and s (6 and 0.2 respectively, in the example above).

The complete set of results of our analysis, including all the differential expression scores and the associated p-values, is available as supplementary material (Table S2).

To apply the paired t-test on these data, fold change was calculated for each miRNA and patient by dividing the tumor signal by the normal signal. In cancer types where more than one patient exists the median of the expression values was used in the fold change calculation. The data was then log-transformed to achieve the normality required by the paired t-test. We note that even after the log-transformation, the hypothesis of normality of this distribution is rejected by the Jarque-Bera test [39].

**Figure 2:**
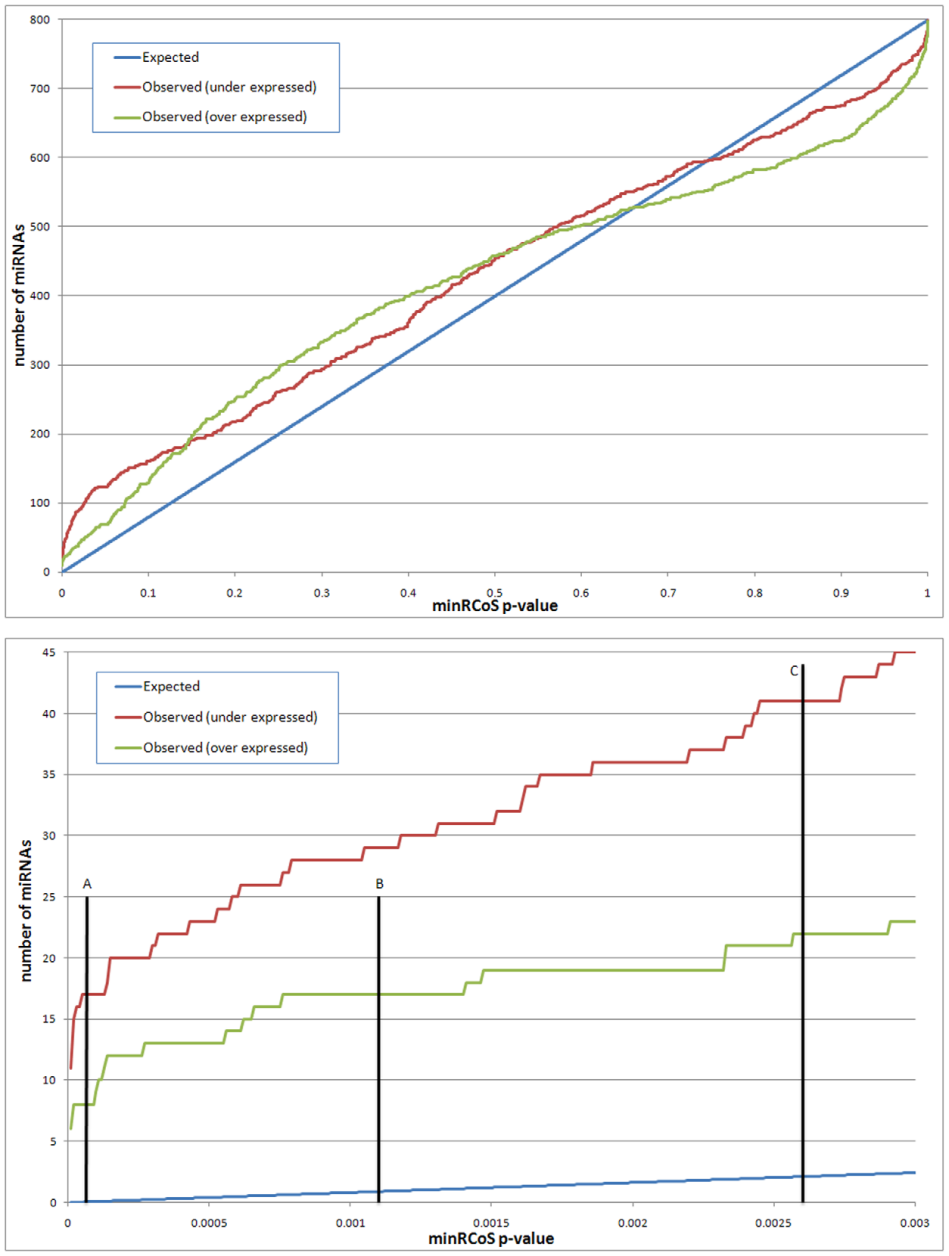
Overabundance analysis of rank consistency. The top plot shows comparison of observed and expected counts of miRNAs for minRCoS p-values. For each p-value (on the x axis), the expected number of miRNA that have this, or better, p-value based on the total number of miRNA on the array, is shown in blue (similar to [54]). The red and green lines symbolize the number of observed miRNAs in our data with these minRCoS p-values. The bottom panel shows a comparison of observed and expected counts of genes with minRCoS p-values of 0.003 or less (a zoom-in on the top panel). Line A indicates the Bonferroni threshold of 0.05, line B indicates the FDR [40] threshold of 0.05 for the over-expressed miRNAs (17 miRNAs) and line C indicates the FDR threshold of 0.05 for the under-expressed miRNAs (41 miRNAs).

The observed and expected numbers of genes for all minRCoS p-values and the levels at which FDR (False Discovery Rate) [40] and Bonferroni of 0.05 are obtained are shown in Figure 2. Note the specific overabundance of differentially expressed miRNAs, as compared to random data expected numbers.

**Figure 3:**
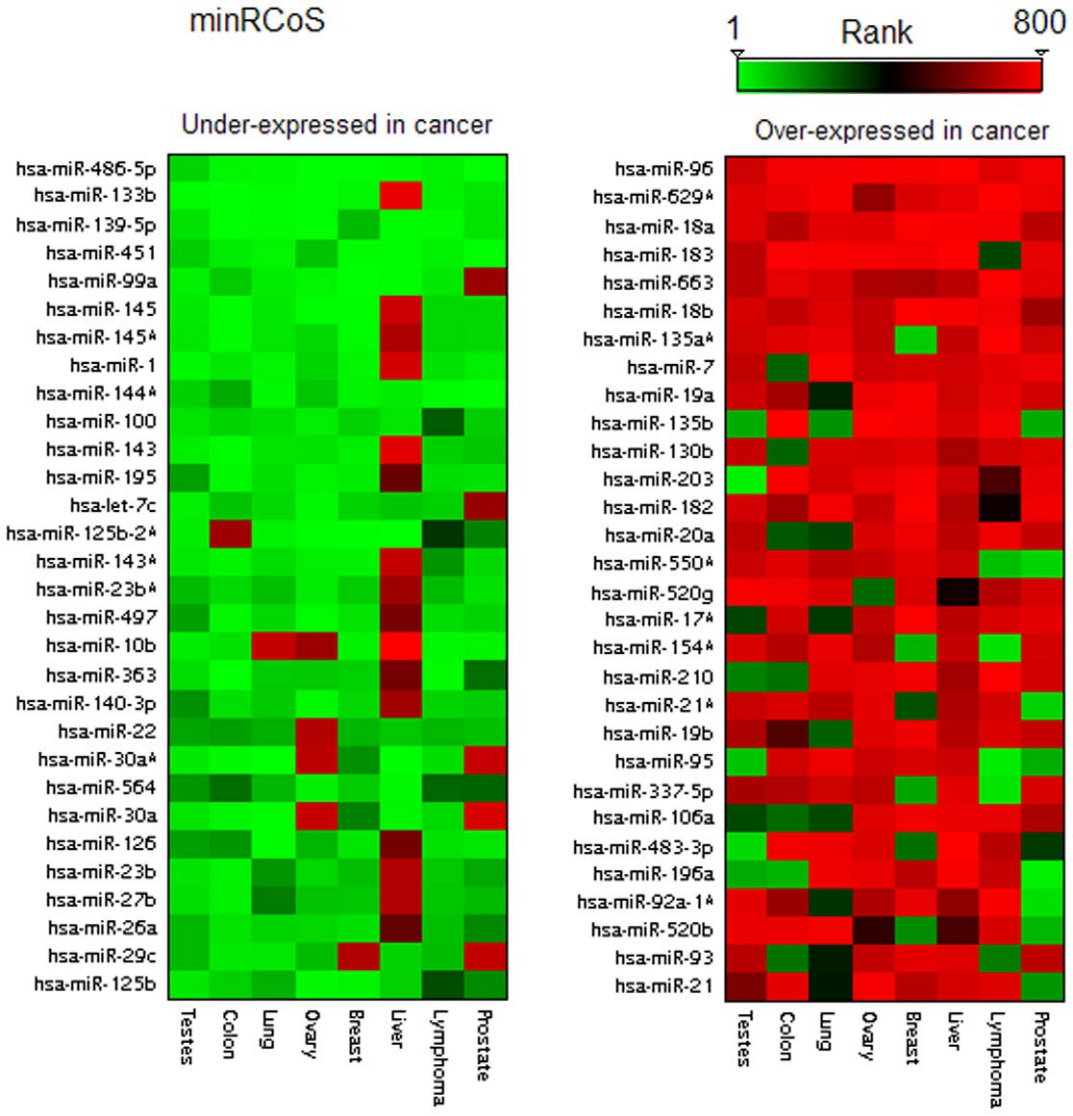
A heatmap of the top ranked miRNAs according to minRCoS analysis. Columns represent cancer types and the rows represent miRNAs. A green entry represents a miRNA with a very high rank i.e. one which is under-expressed in this specific tumor sample compared to the matched normal sample. A red rectangle indicates a miRNA over-expressed in the tumor sample. The left panel shows the top 30 miRNAs universally under-expressed in tumors ranked according to minRCoS analysis and the right panel shows the top 30 miRNAs universally over-expressed in tumors ranked according to minRCoS analysis.

A heatmap of the most significant miRNAs identified by minRCoS analysis is shown in Figure 3. The right panel contains the top 30 miRNAs whose expression levels are consistently increased in cancerous tissues; the left panel contains a list of the top 30 miRNA whose expression levels are consistently decreased in cancerous tissues. Specific conclusions and findings of the analysis are described below, including miRNAs that were not previously universally associated with cancer.

### Differentially Expressed miRNAs Found by RCoS and Not by Other Methods

Some of the miRNAs we observe as differentially expressed were identified as significantly differentially expressed both by matched and by non-matched analysis. For example, miR-96 which is discussed in detail below was found by all four methods described above.

In contrast to miR-96, other miRNAs were detected by minRCoS and not by other methods (both matched and non-matched). An example of such a miRNA which is also not reported in previous multi-type cancer datasets [9], [10] is miR-133b.

**Figure 4:**
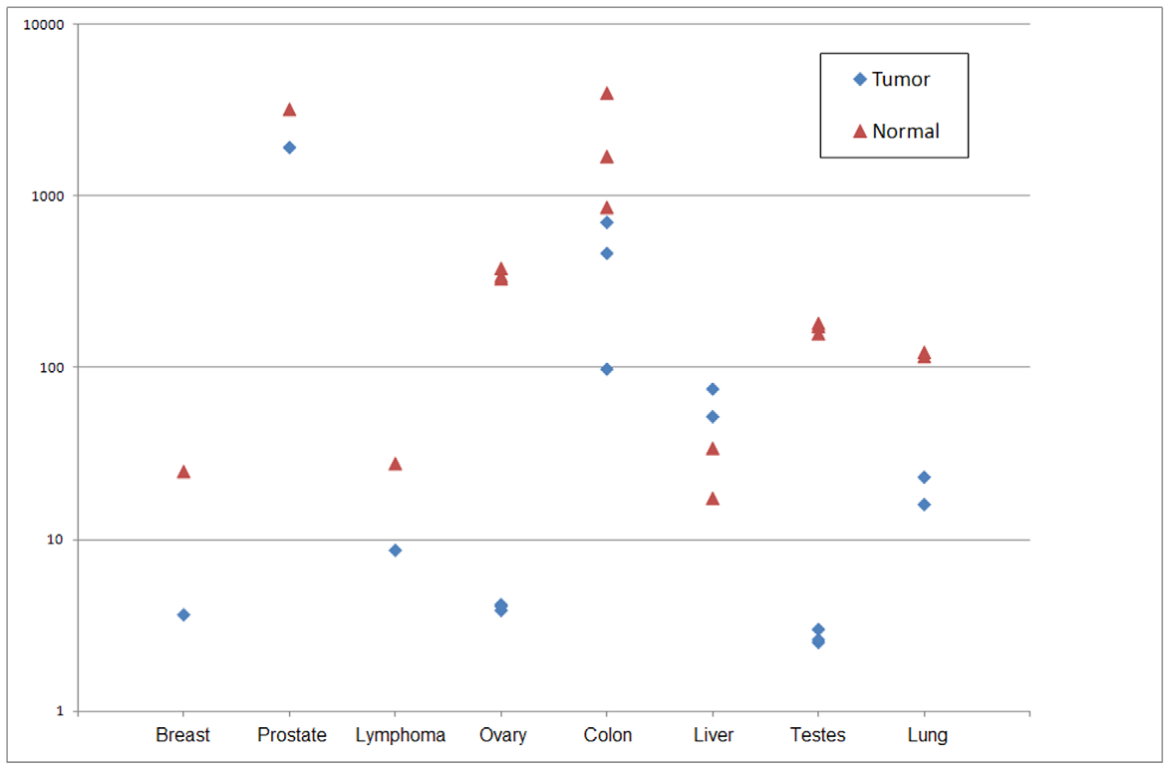
Log signal values of miR-133b. Blue diamonds represent tumor samples and magenta triangles represent normal samples. Note that there is no single threshold that separates all normal samples from all tumor samples. It is also clear that in all but one type (liver) miR-133b is under-expressed in the tumors. This is an example of a differentially expressed miRNA detected by RCoS and not by un-matched analysis nor by paired t-test.

miR-133b receives 7/8 RCoS of 0.048 (p = 5*10^−9^) and a minRCoS p-value of 10^−8^. A close inspection reveals that, excluding the liver sample, miR-133b is under-expressed in all tumor types, compared to the corresponding matched normal tissue. Interestingly (see Figure 4), the miR-133b basal expression values are highly tissue-type variable. Indeed TNoM and t-test do not find a significant separation between the classes. This is an example of the tissue-type variability of miRNA, as noted in the Introduction. miR-133b is also not detectable by paired-test since the paired t-test is greatly affected by the outlier, namely the liver sample. miR-133b was recently found, using RT-PCR, to be consistently down regulated in colorectal cancer by Bandres et al [41]. The authors further show that known proto-oncogenes, like YES1 and MAP3K3, are targeted by miR-133b. We note that since human miR-133a and 133b are highly homologous, differing by only one nucleotide, there could be some cross hybridization in hybridization-based measurements. Cross hybridization in the platform used in our study was shown to be very low by Wang et al. [42], where the authors demonstrate the platform's ability to distinguish between the highly homologous members of the let-7 family.

miR-143 is another example of a miRNA which would not have been found by other methods. When ranking all measured miRNAs using unpaired t-test it ranks as number 70, and when using a paired t-test it ranks as number 59 with a p-value of 0.04. However, when ranking the miRNAs using minRCoS it is ranked as number 11 with a minRCoS p-value of 7*10^−6^ (Figure 3, left panel). miR-143 is known to be under-expressed in several different cancer types as described in [43]-[45], [24].

### miRNAs Over-Expressed in Cancer Compared to Normal

The top ranking over-expressed miRNA in cancer based on minRCoS ranking is miR-96. It is over-expressed in all 8 cancer types and has a minRCoS p-value of 10^−8^. miR-96 was found to be consistently up regulated, validated by RT-PCR, in colorectal cancer [41]. miR-182 and miR-183, which reside in the same cluster with miR-96, on Chr7q32 are both over-expressed in our cancer samples. This leads to the hypothesis that the entire cluster is amplified in cancer. Indeed, Zhang et al [46] show that the locus containing miR-182 is amplified in 28.9% of their ovarian cancer samples. They also state that forced expression of mir-182 in ovarian cancer cell line, significantly promoted tumor growth in vivo, confirming the role of miR-182 as a putative oncogene.

The second top ranking cancer-universal over-expressed miRNA based on minRCoS ranking is miR-629*. It is over-expressed in all 8 cancer types and has a minRCoS p-value of 10^−7^. Little is known about this miRNA, and it was not measured by previous multi-type cancer datasets [9], [10]. Mitchell et al [47] compared miRNA serum levels between 12 mice with human prostate cancer xenografts and 12 controls. They found that mir-629* is greatly over-expressed in the xenograft mice plasma. They therefore hypothesize that miR-629* is potentially secreted from the xenograft cells.

All six members of the mir-17-92 polycistron on chromosome 13 (miR-17, miR-18a, miR-19a, miR-20a, miR-19b-1 and miR-92a-1) are part of the top 30 over-expressed miRNAs. This polycistron is a known oncogene in several cancer types [10], [48], [49].Most members of the miR-17 family (which highly overlaps this cluster) are also in this list.

### miRNAs Under-Expressed in Cancer Compared to Normal

The top ranking under-expressed miRNA in cancer based on minRCoS ranking is miR-486-5p. It is under-expressed in all 8 cancer types and has a minRCoS p-value of 10^−9^. miR-486-5p (along with miR-451 which is also under-expressed in our data) was recently found to be down regulated in Glioblastoma stem cells (CD133+) compared to non-stem (CD133−) cells [50]. CD133+ cells initiate and propagate tumors unlike CD133− cells [51]. miR-133b which is the second top under-expressed miRNA is discussed above.

### Applying Our Methods to a Literature Dataset

We also applied our methods to the Lu et al [9] dataset, as follows. 84 samples from 7 different cancer types (colon, breast, lung, prostate, kidney, bladder and uterus) were used from the Lu et al dataset. These represent all solid tumor types that have at least 2 tumor samples and 2 normal samples. The first 4 types were also measured in our study. For each of the 7 cancer types all 217 miRNAs measured by Lu et al were ranked according to their differential expression between tumor and normal samples, in a given type, using unpaired t-test. We then looked for miRNAs consistently over or under expressed across most tumor types using minRCoS (*r* = 7). The list of all 217 miRNAs measured in the Lu et al study and their p-values is provided in Table S3.

When searching for over-expressed miRNAs we find, for example, consistent high ranks for miR-182 and miR-183(minRCoS p<10^−7^, see Table S3). These miRNAs and their cluster have been previously shown to be over-expressed in cancer and are also detected as such for our dataset, as discussed previously. In addition to detecting the over-expression of miR-182 and miR-183 in Lu's data, we also found more highly concordant results such as significant under-expression of miR-1, miR-195 and miR-99a in both datasets, analyzed using the methods of this study (Table S3).

## Discussion

Our unique dataset, designed to minimize tissue type confounding affects, combined with our novel approach to rank order statistics in discrete random variables enabled us to produce novel findings associating certain miRNAs to universal cancer related processes. Most notably we demonstrate differential expression in a majority of 8 tumor types for miRNAs which were previously only indentified in the context of specific cancer types:

- miR-133b - previously shown to be down regulated in colon cancer [41].
- miR-486-5p - previously shown to be differentially expressed in Glioblastoma [50].
- miR-629* - previously shown to be secreted into the plasma of xenograft bearing mice [47].

The findings of this paper address processes that are common amongst various types of cancer. This is, in some sense, orthogonal and complementary to other miRNA studies [52], [53] that focused on finding differences between different cancer types. miRNA differential expression in multiple cancer types was addressed by Lu et al [9] and Volinia et al [10] as described in the introduction. The current study takes a more statistically refined and accurate approach, providing rigorous statistics and enabling the identification of differentially expressed miRNAs. The study design and our statistical methods allow us to conclude that this differential expression is a reflection of biological state, such as cancer, instead of as reflection of biological identity, such as liver vs. lung.

Traditional approaches to matched-pairs analysis include:

- Paired *t*-Test: *t*-Test applied to the difference between matched measurements.
- Wilcoxon Signed Rank Test: when the differences are not normally distributed, a non parametric method such as a Wilcoxon Signed Rank Test is applied to the differences.
- SAM- statistics implemented within SAM [34] for paired analysis. SAM uses permutation testing to assess score significance.

These approaches suffer from the following shortcomings. The *t*-test is only applicable for normally or close to normally distributed data. In expression data, specifically in miRNA expression, this is often not the case. In addition, the paired t-test requires ranking in each group be performed using fold-change. When ranking genes in each group using a non-paired t-test for example, the paired t-test is no longer applicable. Under the Wilcoxon Signed Rank Test a gene that is always higher in the tumor samples but very slightly so will score better than one that is higher by a large margin in all patients but one and is just slightly lower in that outlier. For example, when ranking all miRNAs in our cohort as discussed in the results section the ranks of miR-133b are <10, 4, 6, 1, 16, 770, 26, 39> and the ranks of miR-582-3p are <345, 355, 368, 205, 356, 218, 357, 95>. Because of the low rank of miR-133b in the liver sample, miR-582-3p will score better when using the Wilcoxon Signed Rank Test. We attribute more biological significance to the differential expression of miR-133b since miR-582-3p has a close to median behavior in all tumor types. The third method discussed, SAM, uses permutation testing to assess score significance and therefore is less applicable for cases with small numbers of patients such as the dataset used in this study. Permutation testing also limits the p-values by the number of permutations performed.

Combinatorial methods for analyzing matched expression data are useful in discovering effects that are not necessarily evident when working with statistical scores that don't take the sample matching into account. Generally, when a gene manifests a robust fold change when comparing two clinically different sets of samples, then the same will hold true when the analysis is performed using the matched structure. The opposite is not true. We identified several miRNAs that are clearly differentially expressed as a result of tumor related processes. These miRNAs could not be identified if one ignores the sample matching information. Determining and statistically assessing the differential expression by comparing expression levels in two different conditions in the same patient serves to offset inter-patient variation that exists in such data. Combinatorial methods have an advantage over parametric methods especially in small sample sets and in studies where we cannot impose model assumptions, such as normality of the underlying distributions.

A good example for the utility of our method is seen when applied to the data generated by Lu et al [9]. In this study the research team profiled the miRNA expression in 334 samples and established a pioneering dataset for the study of global miRNA differential expression in cancer. One of the main conclusions of Lu et al was that the overall expression level of miRNAs is down regulated in tumors relative to the normal samples. Moreover, the miRNAs that were specifically identified by the study as differentially expressed in cancer were shown there to be down regulated in cancer. This apparent absence of miRNAs that are up-regulated in cancer has been challenged by later studies [10], [11]. Since our approach is based on ranks instead of on the actual expression values of miRNAs in each cancer type, the RCoS method also detects miRNAs that are up regulated in cancer, such as miR-182 and miR-183, in Lu et al's data. This example illustrates how RCoS can offset possible biases frequently encountered in the experimental data.

Our statistical methods are not limited to matched samples scenarios, nor to miRNA and cancer. They are applicable to other comparison contexts as well. To be applicable the input data should contain ranking of all elements (such as genes or miRNAs) for each group (such as a patient). This ranking reflects a quantity of interest that was computed or measured in each group, such as the extent of differential expression. The methods will find elements with consistent high ranks across all (or most) of the groups. Software for computing this is available at: http://bioinfo.cs.technion.ac.il/people/zohar/RCoS/

The concordance of the findings of our study with the findings of several other studies and the use of RCoS on the Lu et al data are strongly supportive of the cancer-universal nature of the differential expression of several known cancer-associated miRNAs, namely: miR-133, miR-96 and miR-182. Importantly, this concordance demonstrates the utility of our statistical methodology for analyzing data from different platforms and multiple cancer types. Also, it lends confidence in the miRNAs identified here as differentially expressed in cancer. Thus, in addition to identifying the already known cancer-associated miRNAs mentioned above, our method has identified two novel cancer-associated miRNAs, namely miR-486-5p and miR-629*. As we tested multiple tumor types, these appear to be novel cancer-universal miRNAs.

## Supporting Information

Text S1 Pseudo-Code for computing m/r RCoS and minRCoS
(0.03 MB DOC)

Table S1 Normalized data matrix
(0.52 MB XLS)

Table S2 Table of all miRNAs measured along with their RCoS and minRCoS p-values, t-test and paired t-test
(0.30 MB XLS)

Table S3 Results of applying the RCoS statistics on the Lu et al [9] dataset.
(0.09 MB XLS)

## References

[1] Alizadeh AA, Eisen MB, Davis RE, Ma C, Lossos IS (2000). Distinct types of diffuse large B-cell lymphoma identified by gene expression profiling.. Nature.

[2] Bittner M, Meltzer P, Chen Y, Jiang Y, Seftor E (2000). Molecular classification of cutaneous malignant melanoma by gene expression profiling.. Nature.

[3] Golub TR, Slonim DK, Tamayo P, Huard C, Gaasenbeek M (1999). Molecular Classification of Cancer: Class Discovery and Class Prediction by Gene Expression Monitoring.. Science.

[4] Huang Q, Liu D, Majewski P, Schulte LC, Korn JM (2001). The Plasticity of Dendritic Cell Responses to Pathogens and Their Components.. Science.

[5] Shyamsundar R, Kim Y, Higgins J, Montgomery K, Jorden M (2005). A DNA microarray survey of gene expression in normal human tissues.. Genome Biology.

[6] Chen MM, Ashley EA, Deng DX, Tsalenko A, Deng A (2003). Novel Role for the Potent Endogenous Inotrope Apelin in Human Cardiac Dysfunction.. Circulation.

[7] Sørlie T, Tibshirani R, Parker J, Hastie T, Marron JS (2003). Repeated observation of breast tumor subtypes in independent gene expression data sets.. Proceedings of the National Academy of Sciences of the United States of America.

[8] Griffiths-Jones S, Grocock RJ, van Dongen S, Bateman A, Enright AJ (2006). miRBase: microRNA sequences, targets and gene nomenclature.. Nucl Acids Res.

[9] Lu J, Getz G, Miska EA, Alvarez-Saavedra E, Lamb J (2005). MicroRNA expression profiles classify human cancers.. Nature.

[10] Volinia S, Calin GA, Liu C, Ambs S, Cimmino A (2006). A microRNA expression signature of human solid tumors defines cancer gene targets.. Proceedings of the National Academy of Sciences of the United States of America.

[11] Esquela-Kerscher A, Slack FJ (2006). Oncomirs - microRNAs with a role in cancer.. Nat Rev Cancer.

[12] van Rooij E, Sutherland LB, Qi X, Richardson JA, Hill J (2007). Control of Stress-Dependent Cardiac Growth and Gene Expression by a MicroRNA.. Science.

[13] Thum T, Catalucci D, Bauersachs J (2008). MicroRNAs: novel regulators in cardiac development and disease.. Cardiovasc Res.

[14] Carè A, Catalucci D, Felicetti F, Bonci D, Addario A (2007). MicroRNA-133 controls cardiac hypertrophy.. Nat Med.

[15] Perkins DO, Jeffries CD, Jarskog LF, Thomson JM, Woods K (2007). microRNA expression in the prefrontal cortex of individuals with schizophrenia and schizoaffective disorder.. Genome Biol.

[16] Sonkoly E, Wei T, Janson PCJ, Sääf A, Lundeberg L (2007). MicroRNAs: novel regulators involved in the pathogenesis of Psoriasis?. PLoS ONE.

[17] Hollstein M, Sidransky D, Vogelstein B, Harris CC (1991). p53 mutations in human cancers.. Science.

[18] Oren M (1999). Regulation of the p53 tumor suppressor protein.. J Biol Chem.

[19] Caldas C, Hahn SA, da Costa LT, Redston MS, Schutte M (1994). Frequent somatic mutations and homozygous deletions of the p16 (MTS1) gene in pancreatic adenocarcinoma.. Nat Genet.

[20] Liggett WH, Sidransky D (1998). Role of the p16 tumor suppressor gene in cancer.. J Clin Oncol.

[21] Rocco JW, Sidransky D (2001). p16(MTS-1/CDKN2/INK4a) in cancer progression.. Exp Cell Res.

[22] Ben-Dor A, Lipson D, Tsalenko A, Reimers M, Baumbusch L (2007). Framework for Identifying Common Aberrations in DNA Copy Number Data.. Research in Computational Molecular Biology.

[23] Calin GA, Dumitru CD, Shimizu M, Bichi R, Zupo S (2002). Frequent deletions and down-regulation of micro- RNA genes miR15 and miR16 at 13q14 in chronic lymphocytic leukemia.. Proc Natl Acad Sci U S A.

[24] Michael MZ, O' Connor SM, van Holst Pellekaan NG, Young GP, James RJ (2003). Reduced accumulation of specific microRNAs in colorectal neoplasia.. Mol Cancer Res.

[25] Eis PS, Tam W, Sun L, Chadburn A, Li Z (2005). Accumulation of miR-155 and BIC RNA in human B cell lymphomas.. Proc Natl Acad Sci U S A.

[26] Evans DL, Leemis LM, Drew JH (2006). The Distribution of Order Statistics for Discrete Random Variables with Applications to Bootstrapping.. INFORMS JOURNAL ON COMPUTING.

[27] Rice JA (1995). Mathematical Statistics and Data Analysis.

[28] Ho M, Yang E, Matcuk G, Deng D, Sampas N (2003). Identification of endothelial cell genes by combined database mining and microarray analysis. Physiol.. Genomics.

[29] Chakravarti I, Laha R, Roy J (1967). Handbook of Methods of Applied Statistics.

[30] Hollander M, Wolfe D (1973). Nonparametric Statistical Methods.

[31] Ben-Dor A, Bruhn L, Friedman N, Nachman I, Schummer M (2000). Tissue classification with gene expression profiles.. J Comput Biol.

[32] Hedenfalk I, Duggan D, Chen Y, Radmacher M, Bittner M (2001). Gene-Expression Profiles in Hereditary Breast Cancer.. N Engl J Med.

[33] Hedenfalk I, Ringnér M, Ben-Dor A, Yakhini Z, Chen Y (2003). Molecular classification of familial non-BRCA1/BRCA2 breast cancer.. Proceedings of the National Academy of Sciences of the United States of America.

[34] Tusher VG, Tibshirani R, Chu G (2001). Significance analysis of microarrays applied to the ionizing radiation response.. Proceedings of the National Academy of Sciences of the United States of America.

[35] Zuo F, Kaminski N, Eugui E, Allard J, Yakhini Z (2002). Gene expression analysis reveals matrilysin as a key regulator of pulmonary fibrosis in mice and humans.. Proceedings of the National Academy of Sciences of the United States of America.

[36] Levy AM, Gilad O, Xia L, Izumiya Y, Choi J (2005). Marek's disease virus Meq transforms chicken cells via the v-Jun transcriptional cascade: A converging transforming pathway for avian oncoviruses.. Proceedings of the National Academy of Sciences of the United States of America.

[37] Agilent Feature Extraction Software Manual (n.d.). http://cp.chem.agilent.com/Library/usermanuals/Public/G4460-90019_FE_10.5_User.pdf.

[38] Edgar R, Domrachev M, Lash AE (2002). Gene Expression Omnibus: NCBI gene expression and hybridization array data repository.. Nucl Acids Res.

[39] Jarque CM, Bera AK (1987). A Test for Normality of Observations and Regression Residuals.. International Statistical Review.

[40] Benjamini Y, Hochberg Y (n.d.). Controlling the False Discovery Rate: A Practical and Powerful Approach to Multiple Testing.. http://dx.doi.org/10.2307/2346101.

[41] Bandres E, Cubedo E, Agirre X, Malumbres R, Zarate R (2006). Identification by Real-time PCR of 13 mature microRNAs differentially expressed in colorectal cancer and non-tumoral tissues.. Molecular Cancer.

[42] Wang H, Ach RA, Curry B (2007). Direct and sensitive miRNA profiling from low-input total RNA.. RNA.

[43] Akao Y, Nakagawa Y, Naoe T (2006). MicroRNAs 143 and 145 are possible common onco-microRNAs in human cancers.. Oncol Rep.

[44] Lui W, Pourmand N, Patterson BK, Fire A (2007). Patterns of known and novel small RNAs in human cervical cancer.. Cancer Res.

[45] Slaby O, Svoboda M, Fabian P, Smerdova T, Knoflickova D (2007). Altered expression of miR-21, miR-31, miR-143 and miR-145 is related to clinicopathologic features of colorectal cancer.. Oncology.

[46] Zhang L, Volinia S, Bonome T, Calin GA, Greshock J (2008). Genomic and epigenetic alterations deregulate microRNA expression in human epithelial ovarian cancer.. Proc Natl Acad Sci U S A.

[47] Mitchell PS, Parkin RK, Kroh EM, Fritz BR, Wyman SK (2008). Circulating microRNAs as stable blood-based markers for cancer detection.. Proceedings of the National Academy of Sciences.

[48] He L, Thomson JM, Hemann MT, Hernando-Monge E, Mu D (2005). A microRNA polycistron as a potential human oncogene.. Nature.

[49] Mendell JT (2008). miRiad Roles for the miR-17-92 Cluster in Development and Disease.. Cell.

[50] Gal H, Pandi G, Kanner AA, Ram Z, Lithwick-Yanai G (2008). MIR-451 and Imatinib mesylate inhibit tumor growth of Glioblastoma stem cells.. Biochem Biophys Res Commun.

[51] Singh SK, Hawkins C, Clarke ID, Squire JA, Bayani J (2004). Identification of human brain tumour initiating cells.. Nature.

[52] Rosenfeld N, Aharonov R, Meiri E, Rosenwald S, Spector Y (2008). MicroRNAs accurately identify cancer tissue origin.. Nat Biotech.

[53] Lebanony D, Benjamin H, Gilad S, Li J, Cholakh H (2008). MicroRNAs as a diagnostic tool for differentiating squamous cell lung cancer from other non small cell lung cancers.. J Clin Oncol (Meeting Abstracts).

[54] Ben-Dor A, Friedman N, Yakhini Z (2001). Class discovery in gene expression data. Proceedings of the fifth annual international conference on Computational biology.

