# Supplementary material for: Novel Rank-Based Statistical Methods Reveal MicroRNAs with Differential Expression in Multiple Cancer Types
Source: PLoS One. 2009 Nov 25;4(11):e8003. doi: 10.1371/journal.pone.0008003 (PMC2777376; doi:10.1371/journal.pone.0008003)
Supplement: Text S1 — Pseudo-Code for computing m/r RCoS and minRCoS (0.03 MB DOC) [file pone.0008003.s001.doc]

# Pseudo code for calculating m/r RCoS

**Input:** An N by r ranks matrix. For each patient (or subject or subset) all the genes are ranked according to a specific criterion (such as differential expression). In our case, for each patient, the gene most over-expressed in class A is ranked 1 and the gene most under-expressed is ranked N.

The input matrix should look like this (for r=6):

| .  .  . | .  .  . | .  .  . | .  .  . | .  .  . | .  .  . | .  .  . |
| --- | --- | --- | --- | --- | --- | --- |
| Gene g | 1 | 30 | 47 | 12 | 16 | 5 |
| .  .  . | .  .  . | .  .  . | .  .  . | .  .  . | .  .  . | .  .  . |

**Calculate m/r RCoS for gene g:**

1. Get the relevant row for gene g. This is an array of length r whose members are between 1 and N. We will call this array gRanks.
2. gSortedRanks = sort(gRanks). Now 1≤gSortedRanks[1]≤gSortedRanks[2]≤…≤gSortedRanks[r]≤N.
3. The m/r RCoS for gene g is: .

# Calculating the minRCoS score

1. Calculate the m/r RCoS for each m between 1 and r. Denote this score by S_m_.
2. For each such score calculate its p-value:

$p(m)=p-Val(s_{m},m)=\sum_{k=m}^{r} \left(\begin{matrix} r \\ k \end{matrix}\right)s_{m}^{k}(1-s_{m})^{(r-k)}$

1. The minRCoS score is the minimum of all these p-values: minRCoS = min_1≤m≤r_p(m). (Note that this is a statistical score, not a p-value. See paper for details).
